# Differential HDAC1/2 network analysis reveals a role for prefoldin/CCT in HDAC1/2 complex assembly

**DOI:** 10.1038/s41598-018-32009-w

**Published:** 2018-09-12

**Authors:** Charles A. S. Banks, Sayem Miah, Mark K. Adams, Cassandra G. Eubanks, Janet L. Thornton, Laurence Florens, Michael P. Washburn

**Affiliations:** 1 Stowers Institute for Medical Research, Kansas City, MO 64110 USA; 2 Department of Pathology & Laboratory Medicine, University of Kansas Medical Center, Kansas City, KS 66160 USA

## Abstract

HDAC1 and HDAC2 are components of several corepressor complexes (NuRD, Sin3, CoREST and MiDAC) that regulate transcription by deacetylating histones resulting in a more compact chromatin environment. This limits access of transcriptional machinery to genes and silences transcription. While using an AP-MS approach to map HDAC1/2 protein interaction networks, we noticed that N-terminally tagged versions of HDAC1 and HDAC2 did not assemble into HDAC corepressor complexes as expected, but instead appeared to be stalled with components of the prefoldin-CCT chaperonin pathway. These N-terminally tagged HDACs were also catalytically inactive. In contrast to the N-terminally tagged HDACs, C-terminally tagged HDAC1 and HDAC2 captured complete histone deacetylase complexes and the purified proteins had deacetylation activity that could be inhibited by SAHA (Vorinostat), a Class I/II HDAC inhibitor. This tag-mediated reprogramming of the HDAC1/2 protein interaction network suggests a mechanism whereby HDAC1 is first loaded into the CCT complex by prefoldin to complete folding, and then assembled into active, functional HDAC complexes. Imaging revealed that the prefoldin subunit VBP1 colocalises with nuclear HDAC1, suggesting that delivery of HDAC1 to the CCT complex happens in the nucleus.

## Introduction

Levels of gene expression can be modulated by controlling the acetylation state of lysine residues within unstructured histone N-terminal tails. Such lysine residues are acetylated in transcriptionally active genetic regions by histone acetyltransferases (HATs); removal of the acetyl-lysine marks by histone deacetylases (HDACs) results in chromatin condensation and reduces levels of gene expression^1^. Correct targeting of HATs and HDACs to specific loci at the appropriate time is critical for proper control of gene expression and cellular function^2^. Both HATs and HDACs are assembled into multisubunit protein complexes for targeting to their proper destinations. Specifically, two class I HDACs, HDAC1 and HDAC2, are targeted to genes as components of the NuRD, Sin3, CoREST, and MiDAC complexes^3^. However, how class I HDACs are assembled into these complexes is poorly understood.

In addition to being components of chromatin remodelers, previous evidence suggests that class I HDACs are also components of the CCT interactome^4^. The CCT chaperonin complex can co-operate with cochaperones, such as prefoldin, to complete ATP dependent folding of client proteins^5,6^. Intriguingly, the CCT complex might also promote protein complex assembly. For example, CCT is required for the assembly of folded VHL protein into the VBC tumor suppressor complex^7^ as well as for the assembly of HDAC3 into the SMRT complex^8^.

Correct assembly of HDACs into functional complexes is crucial, as abnormal recruitment of HDAC complexes can have profound effects on gene expression and cellular behavior. For example, in acute myeloid leukemia patients with the chromosomal translocation t(8; 21), the abnormal AML1-ETO fusion protein appears to misdirect HDAC1 containing corepressor complexes to AML1 binding sites, repressing genes needed for myeloid differentiation and resulting in leukemogenesis^9–11^. Understanding the mechanisms of aberrant HDAC recruitment can provide a rationale for clinical treatment. In this case, the erroneous silencing of AML1 regulated genes by misplaced HDAC containing complexes can be reversed using HDAC inhibitors such as valproic acid, allowing transformed cells to differentiate^12,13^. Although this example illustrates one process by which improper targeting of HDACs can cause disease, misregulated HDACs feature in diverse cancer types (tabulated by Li and Seto^14^) and HDAC containing complexes can promote tumorigenesis in different ways^15^. Elucidating how HDACs function in the context of different protein complexes is therefore essential in developing effective disease treatments.

Interactions between HDACs and other components of the protein complexes within which they reside can be mapped using Affinity Purification Mass Spectrometry (AP-MS). AP-MS approaches rely on recombinant affinity tagged bait proteins, which can be expressed in cells for incorporation into endogenous protein complexes, facilitating subsequent capture of these complexes. AP-MS techniques have been invaluable both in mapping the components of different complexes and investigating how protein associations change in different environments. Although the affinity tagged version of the protein can be used to substitute for the endogenous protein and investigate how the endogenous protein might behave, addition of the tag can affect the behavior of the recombinant protein; we have previously shown that even the addition of a single amino acid at the terminus of an amino acid chain can result in spurious protein interactions^16^. Here we show that changing the location of a Halo affinity tag on either HDAC1 or HDAC2 results in a rewiring of the HDAC protein interaction network. The Halo tag is a modified version of a haloalkane dehalogenase, which becomes covalently attached to haloalkane ligands^17^. This allows isolation and purification of Halo-tagged fusion proteins and their interaction partners using immobilized ligands, as well as imaging of Halo-tagged proteins using fluorescent ligands. N-terminally tagged versions of HDAC1 and HDAC2 have minimal enzyamtic activity and preferentially retained by prefoldin and the chaperonin complex CCT, whereas C-terminally tagged versions of HDAC1 and HDAC2 are enzymatically active and assemble into the histone deacetylase complexes CoREST, Sin3, NuRD, and MiDAC. Hence, the positioning of the affinity tag on the N-terminus of HDAC1 or HDAC2 traps intermediate complexes on the pathway towards active chromatin complex assembly. Guenther and coworkers^8^ previously proposed a model in which HDAC3 is primed by Hsc70 and the CCT complex before being used in functional HDAC3 complexes. However, in contrast to HDAC3, the prefoldin and CCT complexes coordinate assembly of HDAC1 and HDAC2 into active chromatin associated complexes.

## Results

**Figure 1:**
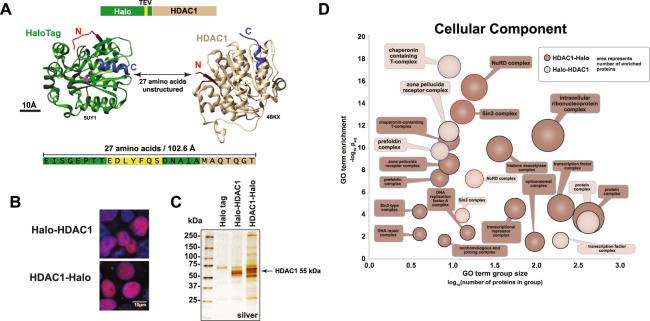
Protein complexes associated with Halo-tagged HDAC1 expressed in HeLa cells. (**A**) Both the N and C termini of HDAC1 are potentially available for affinity tagging. The structures of HaloTag (green, ligand binding site in pink, PDB ID 5UY1^60^) and human HDAC1 (brown, PDB ID 4BKX^61^) visualized using Chimera^62^ are shown to scale. The 27 amino-acid region in Halo-HDAC1 not present in the structures is also shown to scale. An additional 106 amino acids at the C terminus of HDAC1 are absent from the structure. (**B**) Localization of Halo-HDAC1 and HDAC1-Halo in HEK293T cells. Halo-tagged proteins are labeled with TMRDirect™ ligand (red). Nuclei are stained with Hoechst dye (blue). (**C**) Proteins enriched by either Halo-HDAC1 or HDAC1-Halo in HeLa cells. Proteins were resolved on a 4–15% SDS-PAGE gel and visualized by silver staining. Similar samples were analysed by Western blotting using HDAC1 antibodies to confirm the presence of full length HDAC1 in the eluates (Supplementary Figure 1). (**D**) Protein complexes copurifying with Halo-HDAC1 or HDAC1-Halo. Proteins significantly enriched (log_2_FC > 2, FDR_up_ < 0.05) in Halo-HDAC1 or HDAC1-Halo purifications (3 biological replicates each) compared with controls expressing the Halo tag alone (3 biological replicates) were analysed for enriched GO terms (cellular component) using DAVID^32^ (Supplementary Table S2). Significantly enriched GO terms (p_adj_ < 0.05) which include the word “complex” are shown.

To investigate protein complexes associating with human HDAC1 using Halo affinity chromatography, we first examined the structure of HDAC1 to assess whether either terminus of HDAC1 appeared to be accessible for tagging. Neither the N-terminus nor the C-terminus of HDAC1 appeared inaccessible (Fig. 1A). In addition, the vectors used to engineer Halo tags provide unstructured linker regions, which might mitigate possible steric hindrance by the tag of HDAC1 binding to its partner proteins. For example, Halo-HDAC1 (pFN21A) has 20 exogenous amino acids between the structured region of the Halo tag and the HDAC1 protein, which in turn has a further seven amino acids at the N-terminus not seen in the HDAC1 crystal structure. If unstructured, these 27 amino acids would have a calculated contour length of 102.6 Å^18^ (shown to scale with the structures of the Halo tag and HDAC1 in Fig. 1A). The C-terminal 106 amino acids of HDAC1 are also absent from the HDAC1 crystal structure. Having confirmed that both HDAC1 termini appeared accessible, we next looked at 13 published studies that had used tagged versions of HDAC1 (or its homolog HDAC2) for investigations and we saw that both termini had previously been used for affinity tagging^19–31^ (Supplementary Table S1). Intriguingly, two of these studies noted that HDAC1 behaviour was modulated by affinity tag presence. First, Taplick *et al*. found that although N-terminally tagged mouse GST-HDAC1 interacted with untagged HDAC1 *in vitro*, N-terminally tagged HA-HDAC1 expressed in 3T3 fibroblasts failed to homodimerize with endogenous HDAC1^26^. They also found that although HA-HDAC1 correctly localized to the nucleus, purified HA-HDAC1 had a significantly lower deacetylase activity with purified histones than HDAC1-myc. Second, a later study by Li *et al*. had found that removal of an N terminal His tag from nickel affinity purified preparations of human His-HDAC1 resulted in increased HDAC activity^28^. These previous reports suggested that appending an affinity tag might affect the behavior of recombinant HDAC1, although how the presence of the tag might affect HDAC1 assembling into cellular complexes remained uncertain. We therefore decided to characterize both Halo-HDAC1 and HDAC1-Halo using affinity purification mass spectrometry (AP-MS) experiments.

### The location of a Halo affinity tag influences HDAC1 association with endogenous protein complexes in HeLa cells

Consistent with the evidence reported by Taplick *et al*. that the location of an affinity tag does not affect mouse HDAC1 localization, we found that both Halo-HDAC1 and HDAC1-Halo localize to HEK293T cell nuclei (Fig. 1B). To identify HDAC1 interaction partners, we next affinity purified protein complexes using Halo-HDAC1 and HDAC1-Halo expressed in HeLa cells and first analysed the copurifying proteins by SDS PAGE and silver staining (Fig. 1C). Although both Halo-HDAC1 and HDAC1-Halo preparations contained factors not present in the control, there appeared to be distinct differences between the populations of proteins detected with each HDAC1 bait. To identify these sets of proteins we analysed replicates of each purification by MudPIT and detected 58 Halo-HDAC1 and 218 HDAC1-Halo associated proteins (log_2_FC > 2, FDR_up_ < 0.05). We then asked whether these two sets of proteins were enriched for components of known protein complexes using the DAVID bioinformatics resource^32^ (Supplementary Table S2 and Fig. 1D). Both purifications contained components of: the chaperonin containing T (CCT) complex (CCT subunits are also present in the zona pellucida receptor complex); the prefoldin complex; and the histone deacetylase complexes Sin3 and NuRD. In addition, we noticed: first, that more subunits of Sin3 and NuRD were captured by HDAC1-Halo than by Halo-HDAC1 (Fig. 1D, compare size of dark brown to light brown circles with red borders); second, that although similar numbers of CCT and prefoldin subunits were captured by each bait, the enrichment of CCT and prefoldin by Halo-HDAC1 was statistically more significant by HDAC1-Halo (Fig. 1D, compare adjusted p values of circles with blue borders).

### Quantitative rewiring of the HDAC1 protein complex interaction network after tag relocation

**Figure 2:**
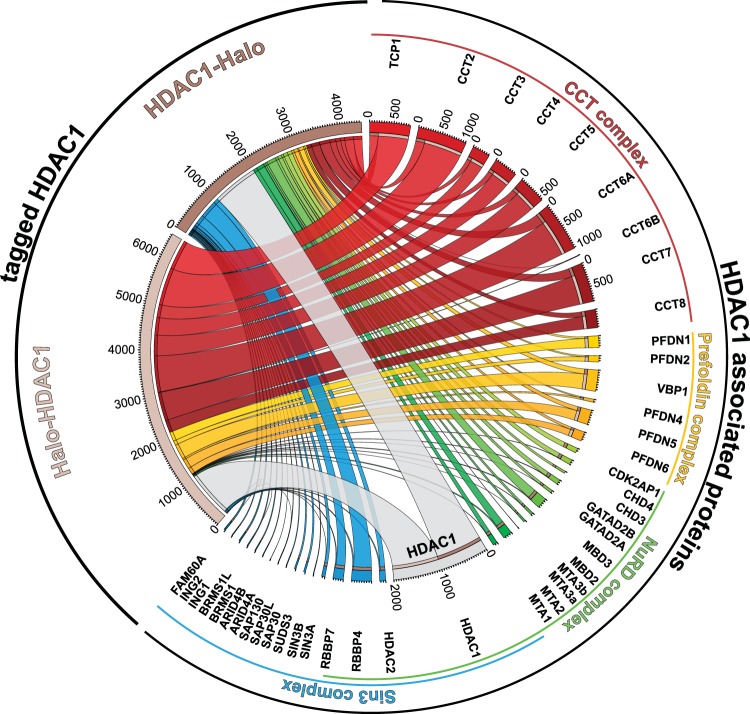
Quantitative comparison of chaperone/chaperonin and histone deacetylase complexes associated with HeLa cell expressed Halo-HDAC1 and HDAC1-Halo. Components of the complexes CCT (red ribbons), prefoldin (yellow ribbons), NuRD (green ribbons) and Sin3 (blue ribbons) captured by either Halo-HDAC1 or HDAC1-Halo fusion proteins (grey ribbons) were quantitated by calculating dBNSAF values (Supplementary Table S3). Values 1000 x dBNSAF have been visualized using Circos^63^.

Of course, these lower adjusted p values for CTT/prefoldin enrichment with Halo-HDAC1 compared with HDAC1-Halo might simply arise because fewer total proteins were enriched by Halo-HDAC1 (58 compared with 218). To ask whether there are quantitative differences between the amounts of each subunit of the CCT, prefoldin, Sin3, and NuRD complexes captured by either Halo-HDAC1 or HDAC1-Halo, we used a spectral counting approach to calculate dBNSAF values^33^ for each subunit of these complexes, and then compared corresponding values for subunits captured by either Halo-HDAC1 or HDAC1-Halo (Fig. 2, Supplementary Table S3). The dBNSAF values normalize the distributed spectral abundance factor (dSAF) of each complex subunit to the dSAF of the HDAC1 bait protein for each replicate to account for variations in the amount of bait protein expressed in each replicate experiment. Halo-HDAC1 captured relatively larger amounts of each detectable CCT and prefoldin complex subunit than HDAC1-Halo (Fig. 2 red and yellow ribbons). In contrast HDAC1-Halo captured relatively larger amounts of each detectable NuRD and Sin3 subunit (Fig. 2 green and blue ribbons).

### Tag mediated modulation of HDAC association with Sin3, NuRD, CCT, and prefoldin complexes also occurs with HDAC2 or with HDAC1 expressed in HEK293T cells

**Figure 3:**
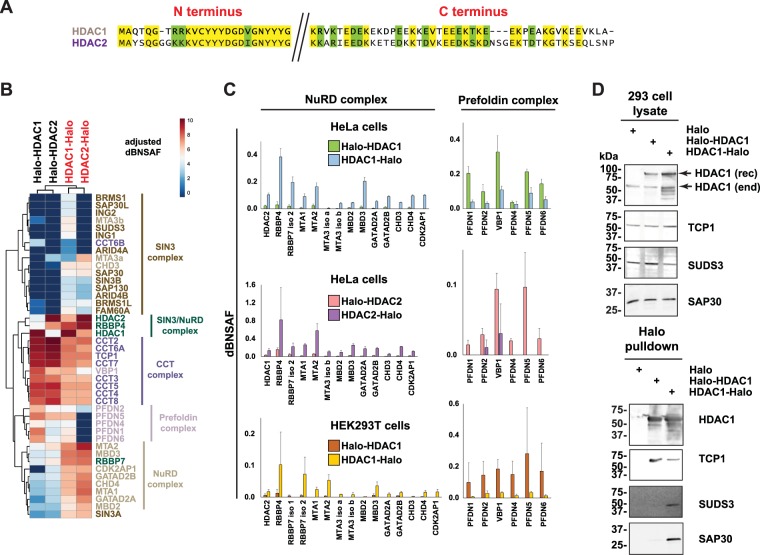
HDAC2 (HeLa) or HDAC1 (HEK293T) associations with Sin3, NuRD, CCT and prefoldin complexes depends on affinity tag location. (**A**) Sequence alignment of the N and C termini of human HDAC1 (NP_004955) and human HDAC2 (NP_001518), using the AlignX tool in Vector NTI^64^. Identical residues are highlighted in yellow and similar residues in green. (**B**) Hierarchical clustering of Halo-tagged HDAC1 and HDAC2 associated complexes. Bait normalized dNSAF values (Supplementary Tables S3 and S4) were scaled 1000x prior to clustering using R. Values were further adjusted using a log_2_ transformation for representation using the indicated colour scale. Dissimilarity matrix calculations were based on Euclidean distance. (**C**) Components of the NuRD or prefoldin complexes copurifying with tagged versions of either HDAC1 (HeLa cells), HDAC2 (HeLa cells) or with HDAC1 (HEK293T cells). Error bars indicate standard deviation. (**D**) Lysates from HEK293T cells transfected with constructs expressing either Halo tag alone, Halo-HDAC1 or HDAC1-Halo were processed as described in “Methods”. Halo purified samples were analysed using SDS-PAGE and visualised by Western blotting using antibodies to the proteins indicated. Bands corresponding to recombinant Halo-tagged HDAC1 (HDAC1(rec)) or endogenous HDAC1 (HDAC1(end)) in the lysate samples are marked. Full length images of Western blots are presented in Supplementary Figure 2.

Having observed that Halo-HDAC1 and HDAC1-Halo associate differently with endogenous HeLa cell histone deacetylase and chaperonin complexes, we next investigated whether similar changes in complex association might occur in additional similar experiments. First, we investigated tagged versions of the HDAC1 homologue HDAC2, and second, we investigated tagged versions of HDAC1 expressed in a different cell type, HEK293T cells (Fig. 3). HDAC2 is an HDAC1 homologue with modest sequence differences from HDAC1 at each end of the amino acid chain (Fig. 3A). We used Halo-HDAC2 and HDAC2-Halo to capture protein complexes from HeLa cells as we had done for HDAC1 and quantified the relative amounts of Sin3, NuRD, CCT, and prefoldin, complex subunits in each purification by calculating dBNSAF values as before. We then used hierarchical clustering analysis to compare these dBNSAF values with the corresponding values calculated for Halo-HDAC1 and HDAC1-Halo purified complexes (Fig. 3B, Supplementary Tables S3 and S4). Notably, most unique components of these four complexes clustered together (as did 3 of the 4 shared Sin3/NuRD subunits). In addition, the baits Halo-HDAC1 and Halo-HDAC2 clustered together, and the baits HDAC1-Halo and HDAC2-Halo clustered together. This suggests that the tagged versions of HDAC2 are behaving similarly to those of HDAC1 in capturing protein complexes differently depending on the location of the Halo tag. Finally, as networks of protein interactions can vary according to cell type^34,35^, we asked whether the affinity tag dependent changes in HDAC1 association with HeLa cell complexes also occurred in HEK293T cells. As we had seen previously in experiments using tagged versions of either HDAC1 or HDAC2 in HeLa cells, relatively larger amounts of NuRD complex subunits copurified with C terminally tagged HDAC1-Halo in HEK293T cells compared with the amounts copurifying with N-terminally tagged Halo-HDAC1 (Fig. 3C, Supplementary Tables S3–S5). Furthermore, relatively smaller amounts of the prefoldin complex subunits copurified with HDAC1-Halo than with Halo-HDAC1 in HEK293T cells (Fig. 3C). Western blotting analysis also confirmed that HEK293T cell expressed Halo-HDAC1 associates with CCT complex subunit TCP1 but the Sin3 subunits SUDS3 and SAP30 were barely detectable, whereas HDAC1-Halo clearly associates with SUDS3 and SAP30 (Fig. 3D). Taken together, the results of Figs 1–3 support that the N- and C-terminally tagged versions of HDAC1 and HDAC2 exist preferentially associated with either molecular chaperones (CCT and prefoldin) for N-terminally tagged fusion proteins or assembled into histone deacetylase complexes (Sin3 and NuRD) for C-terminally tagged fusion proteins.

### Changing the affinity tag location rewires the HDAC1/HDAC2 protein interaction network

**Figure 4:**
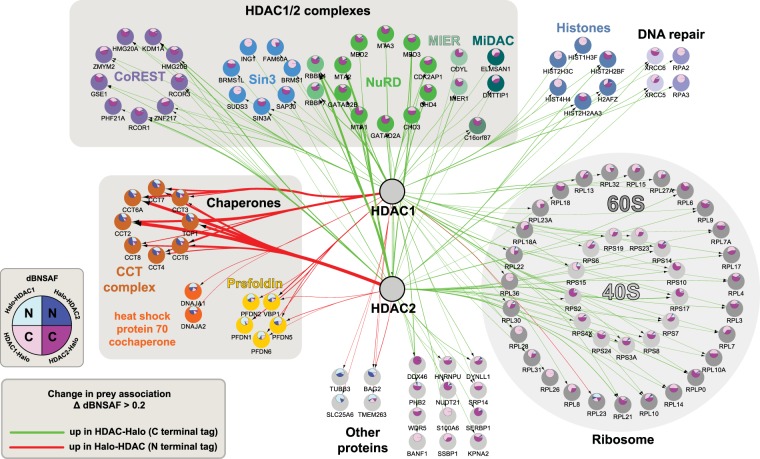
Rewiring of a HeLa cell HDAC1/2 protein interaction network following Halo affinity tag relocation. Differential network analysis was performed using Diffany^36^ to compare HDAC1/2 interaction networks resulting from HDAC purifications using either N-terminal or C-terminal Halo affinity tags. Network analysis was performed on the set of proteins significantly enriched with at least one of the bait proteins Halo-HDAC1, Halo-HDAC2, HDAC1-Halo, or HDAC2-Halo (QSPEC log_2_ FC > 2, QSPEC p_adj_ < 0.05, detected > 50% replicates). The differential network highlights changes in values for dBNSAF between Halo-HDAC and HDAC-Halo, with a minimum change of 0.2. The edge width (unbundled regions) is proportional to ΔdBNSAF and the edge color indicates the direction of change (green = increased values in HDAC-Halo samples, red = increased values in Halo-HDAC samples). Pie charts within nodes indicate the relative distribution of dBNSAF values for HDAC1/2 baits using N-terminal tags (blue segments) and using C-terminal tags (pink/purple segments). An expanded explanation of this differential interaction network analysis can be found in Supplementary Figure 3.

Having investigated how the location of the affinity tag specifically affects HDAC association with the CCT, prefoldin, Sin3 and NuRD complexes, we explored whether the tag location similarly influenced HDAC1/2 involvement both with other HDAC complexes and with other cellular proteins more generally. We initially generated two protein interaction networks: first, a Halo-HDAC (N terminal tag) network of proteins significantly enriched with either Halo-HDAC1 or Halo-HDAC2; second, a HDAC-Halo (C terminal tag) network of proteins significantly enriched with either HDAC1-Halo or HDAC2-Halo (log_2_FC > 2, p_adj_ < 0.05). To examine the differences between these two networks, we used the bioinformatics tool Diffany^36^ to generate the differential protein interaction network shown in Fig. 4. This differential network indicates proteins with changes in dBNSAF values greater than 0.2 between the “N terminal tag” and “C terminal tag” networks. The widths of the unbundled edge regions in the differential network are proportional to changes in dBNSAF. Proteins with red edges are preferentially enriched in the “N terminal tag” network and those with green edges are preferentially enriched in the “C terminal tag” network (Supplementary Figure 3 illustrates how the differential network is generated). As we had already observed, the differential network shows that C-terminally tagged HDACs preferentially capture the Sin3 and NuRD complexes, whereas the N-terminally tagged HDACs preferentially capture the CCT and prefoldin complexes. In parallel with these earlier observations, C-terminally Halo tagged versions of HDAC1 and HDAC2 preferentially captured subunits of the HDAC complexes CoREST, MEIR and MiDAC, whereas N-terminally tagged versions of these proteins preferentially captured the heat shock protein 70 cochaperone proteins DNAJA1 and DNAJA2 (Fig. 4). We also found many ribosomal proteins preferentially enriched in purifications using C terminally tagged HDACs. Interestingly, the C-terminally tagged bait proteins also preferentially captured histones and DNA repair proteins—it is possible that these proteins are captured via HDAC complex interactions with chromatin.

### HDAC1 association with components of the prefoldin-mediated CCT folding pathway

**Figure 5:**
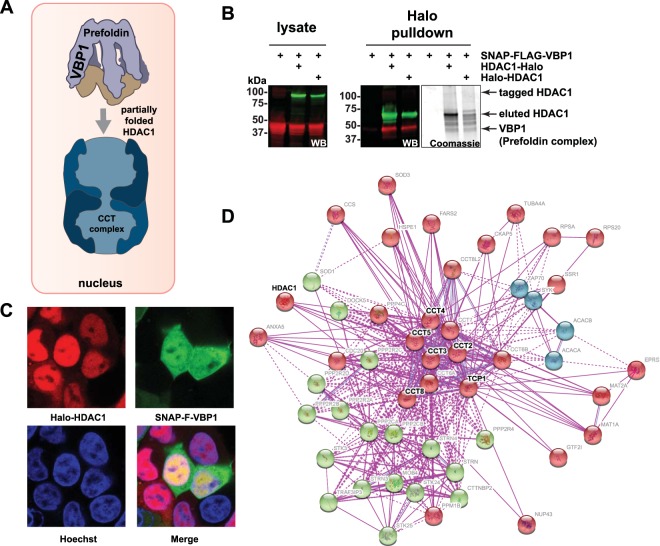
HDAC1 associates with components of the prefoldin-mediated CCT folding pathway. (**A**) The established mechanism for prefoldin delivery of client proteins to the CCT complex^37,44,65^ used to model possible HDAC1 processing by the prefoldin/CCT pathway in the nucleus. (**B**) Prefoldin subunit VBP1 interacts with HDAC1. HEK293T cell lysates expressing tagged VBP1 with or without Halo-tagged versions of HDAC1 were used for Halo affinity purification. Samples were analysed by SDS PAGE followed by Western blotting or Coomassie staining. SNAP-FLAG-VBP1 was detected using anti-FLAG mouse monoclonal primary antibody and IRDye® 680LT labeled goat anti-Mouse secondary antibody. Halo-tagged HDAC1 was detected using anti-HDAC1 rabbit polyclonal primary antibody and IRDye® 800CW labeled goat anti-Rabbit secondary antibody. Full length images are presented in Supplementary Figure 3. (**C**) VBP1 colocalises with HDAC1 in the nucleus. Flp-In™-293 host cells stably expressing Halo-HDAC1 were transiently transfected with SNAP-FLAG-VBP1 and proteins imaged using HaloTag® TMRDirect™ ligand (Halo-HDAC1; red), SNAP-Cell® 505-Star ligand (SNAP-FLAG-VBP1; green), and Hoechst dye (nuclei; blue). (**D**) TCP1 and other CCT complex components interact with HDAC1. The STRING^38^ network showing established TCP1 (CCT complex) interacting proteins was generated using the following settings: minimum required interaction score - medium confidence (0.4); maximum number of interactors - 50; active interaction source – Experiments; clustering method – kmeans (default settings). Clusters are indicated by node color.

Our AP-MS analyses suggested interactions between HDAC1 and components of the prefoldin/CCT complex folding pathway. Prefoldin is a cochaperone that delivers partially folded client proteins to the chaperonin complex CCT for ATP dependent completion of folding to the native state^37^. Since Halo-HDAC1 associates with both prefoldin and CCT complexes and is localized to the nucleus, we hypothesized that prefoldin was transferring partially folded Halo-HDAC1 to the CCT complex in the nucleus (Fig. 5A). If HDAC1 was processed as a CCT complex client protein, CCT might bind to recombinant Halo-HDAC1 but not complete folding. This would explain the higher association of Halo-HDAC1 with prefoldin/CCT components compared with HDAC1-Halo, which once processed would be available for incorporation into HDAC complexes. To gain additional evidence that prefoldin indeed binds HDAC1, we tested the ability of Halo-HDAC1 or HDAC1-Halo to bind a SNAP-tagged version of prefoldin subunit VBP1 in HEK293T cells (Fig. 5B and Supplementary Figure 4). Both tagged versions of HDAC1 captured SNAP-FLAG-VBP1 supporting that HDAC1 binds prefoldin (Fig. 5B). In addition, SNAP-FLAG-VBP1 captured both tagged versions of HDAC1 (Supplementary Figure 4). As we had originally observed both Halo-HDAC1 and HDAC1-Halo in the nucleus, we thought that the apparent accumulation of prefoldin with Halo-HDAC1 (supported by our AP-MS data) would also occur in the nucleus. To test this, we transfected a 293-FRT cell line stably expressing Halo-HDAC1 with SNAP-FLAG-VBP1 for imaging (Fig. 5C). Consistent with the observation that Halo-HDAC1 localised to the nucleus in HEK293T cells (Fig. 1B), 293-FRT cell expressed Halo-HDAC1 was also nuclear. Consistent with the known role of prefoldin/CCT in processing actin and tubulin, we observed significant cytoplasmic SNAP-FLAG-VBP1. In addition, we also observed nuclear SNAP-FLAG-VBP1, particularly in cells expressing higher levels of Halo-HDAC1 (Fig. 5C). This supports that prefoldin delivers HDAC1 to the CCT complex in the nucleus. Finally, to buttress our evidence for HDAC1 as a CCT client protein, we searched the STRING database^38^ for the top 50 interactors with TCP1, a key CCT complex subunit (Fig. 5D). The resulting TCP1 interaction network indicated that there is existing experimental evidence for interactions between HDAC1 and TCP1, as well as between HDAC1 and several other CCT complex subunits (CCT2, CCT3, CCT4, CCT5, and CCT8). Taken together, the results of Fig. 5 support a role for CCT complex processing HDAC1 as a client protein in the nucleus.

### Affinity tag modulation of HDAC1/2 deacetylase activity

**Figure 6:**
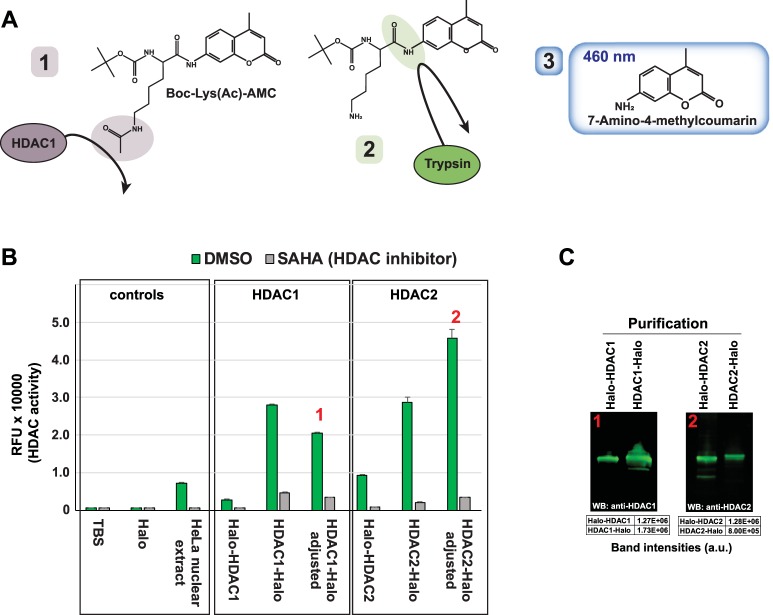
HDAC1/2 deacetylase activity depends on the affinity tag location. (**A**) Substrate used to test HDAC activity. Boc-Lys(Ac)-AMC contains an acetylated lysine moiety that can be deacetylated by class I HDACs (1). Deacetylated substrate is recognized by trypsin (2), and subsequent trypsin digestion releases fluorescent 7-Amino-4-methylcouramin which emits light detectable at 460 nm (3). The structure of Boc-Lys(Ac)-AMC has been reproduced using data from National Center for Biotechnology Information PubChem Compound Database; CID = 9846360, https://pubchem.ncbi.nlm.nih.gov/compound/9846360 (accessed Apr. 17, 2018). (**B**) Activity of HDAC1/2 purified samples. HDAC activity assays were performed as described in Methods using equal volumes of eluate from the indicated purifications. Values are the mean of three technical replicates. Error bars represent standard deviation. Adjusted values account for differences in HDAC1/2 concentration between Halo-HDAC1 and HDAC1-Halo samples, or between Halo-HDAC2 and HDAC2-Halo samples determined by quantitative Western blotting using antibodies to either HDAC1 or HDAC2. (**C**) Relative amounts of HDAC1 or HDAC2 in Halo affinity purifications. Equal volumes of eluate from the indicated Halo affinity purifications were analysed by SDS PAGE and Western blotting. Purified HDAC1 or HDAC2 was detected using rabbit anti-HDAC1 or rabbit anti-HDAC2 primary antibody and IRDye® 800CW labeled goat anti-Rabbit secondary antibody. Fluorescently labeled secondary antibodies were detected using a Li-Cor Odyssey infrared imaging system. Band intensities were quantitated using Image Studio (version 2.1) software (Li-Cor). Full length images are presented in Supplementary Figure 5.

If the CCT complex is unable to complete processing of the Halo-HDAC1 protein, we reasoned that the enzymatic activity of Halo-HDAC1 might consequently be compromised compared with HDAC1-Halo which appears to be used in nuclear HDAC complexes. To test the deacetylation activity of Halo-HDAC1 and HDAC1-Halo, we used the substrate Boc-Lys(Ac)-AMC^39^. In brief, the substrate contains a Boc-protected acetylated lysine residue attached to a C-terminal 7-amino-4 methylcouramin (AMC) moiety (Fig. 6A). If the lysine is deacetylated by HDAC1 (Fig. 6A-1), the substrate can be cleaved with trypsin, releasing fluorescent AMC (Fig. 6A-2 and A-3). Reactions containing HeLa cell nuclear extract generate a fluorescent signal, whereas reactions containing both HeLa nuclear extract together with SAHA, a class I and II HDAC inhibitor, generate minimal signal (Fig. 6B – controls). We tested the activity of samples from Halo-HDAC1 and HDAC1-Halo purifications prepared from equal numbers of transfected HEK293T cells. Samples purified from cells expressing HDAC1-Halo had remarkably greater activity than samples from Halo-HDAC1 expressing cells (Fig. 6B, HDAC1 panel). To ensure that the greater activity of the HDAC1-Halo samples was not solely due to greater expression of the recombinant HDAC1 in these cells, we measured the levels of HDAC1 in each sample by quantitative Western blotting and adjusted the HDAC1-Halo signal to account for differences in the HDAC1 concentration in the Halo-HDAC1 and HDAC1-Halo purified samples (Fig. 6C). After adjustment, we again observed remarkably greater HDAC activity in the samples purified with the C terminally tagged version of HDAC1. To strengthen the evidence that the C-terminally tagged version of the HDAC protein copurifies with greater activity, we also measured the HDAC activity of samples purified from cells expressing either Halo-HDAC2 or HDAC2-Halo. Again, we detected appreciably more HDAC activity in HDAC2-Halo purified samples than in Halo-HDAC2 purified samples (Fig. 6B, HDAC2 panel). In summary, the N terminally Halo tagged HDAC1 and HDAC2, which remain associated with prefoldin/CCT complexes, have minimal HDAC activity, whereas the C terminally tagged HDAC1 and HDAC2, which are built into histone deacetylase complexes have substantial HDAC activity.

## Discussion

We have been able to isolate different populations of HDAC1 bound preferentially to either the CCT complex or assembled into active histone deacetylase complexes depending on the placement of the Halo affinity tag used for isolation. One explanation for this finding is that HDAC1 is processed by the CCT complex before being incorporated into HDAC complexes such as Sin3 and NuRD, and that the placement of an affinity tag at the N terminus of HDAC1 interrupts this process. In essence, placing the affinity tag on the N-terminus of HDAC1 or HDAC2 traps intermediate complexes on the pathway to active and chromatin associated HDAC complexes.

**Figure 7:**
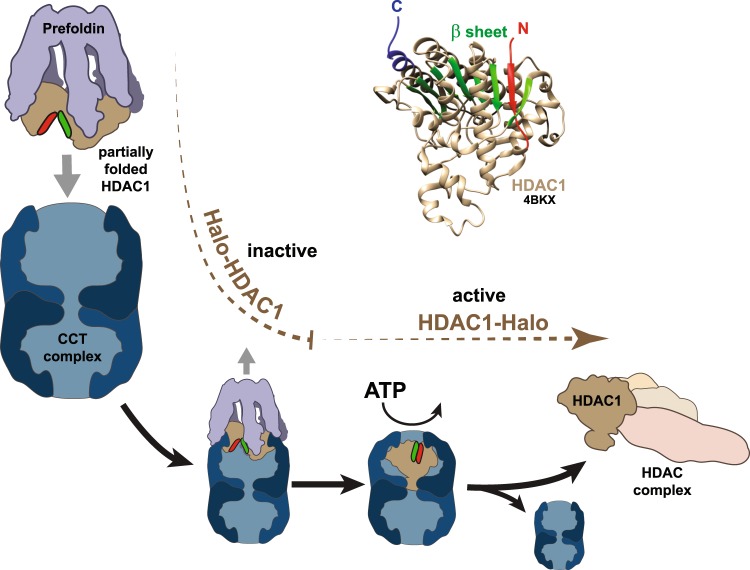
Model for HDAC1 relationship with CCT and HDAC complexes. Previously, Guenther *et al*.^8^ proposed that HDAC3 is primed by binding to the CCT complex prior to binding the corepressor SMRT with release of CCT. Similarly, we suggest that HDAC1 might associate with CCT for ATP-dependent folding^47^ prior to assembly into Sin3, NuRD or CoREST complexes, with this process influenced by the presence of an N-terminal Halo tag. In parallel with previous evidence that CCT binds to and facilitates folding of two β strands within the VHL protein^66^, the CCT complex might facilitate folding of the β sheet within HDAC1 (indicated by green ribbons in the HDAC1 structure (PDB 4BKX^61^, visualized using Chimera^62^) with the N-terminal β strand shown in red); an N-terminal Halo tag might prevent process of HDAC1 folding or of HDAC1 release by CCT for incorporation into HDAC complexes.

Evidence from previous studies supports processing of HDAC1/2 by CCT. First, HDAC1 appears to be part of the CCT interactome. A geneome-wide CCT interaction network defined in yeast identified significant physical and genetic interactions between yeast CCT and yeast histone deacetylase complexes Rpd3 and Set3^40^. In addition, Yam and coworkers identified class I histone deacetylase HDAC3 as one of 170 CCT substrates by screening a mouse cDNA library^41^. HDAC1 has also been listed among 76 proteins that intersect both human and yeast CCT interactomes^4^. Second, previous studies aimed at defining the HDAC1 interactome have identified components of prefoldin and CCT copurifying with HDAC1^20,42^ but the functional significance of this was not explored. Third, Guenther and coworkers previously found evidence that the CCT complex works in concert with Hsc70 to prime HDAC3 prior to its assembly into SMRT corepressor complexes^43^. We propose that HDAC1 and HDAC2 might similarly interact with CCT complex prior to assembly into Sin3, NuRD, CoREST, or MiDAC corepressor complexes (Fig. 7). In contrast to HDAC3, we propose that HDAC1 and HDAC2 are delivered to CCT by prefoldin.

The mechanism of delivery of client proteins to the CCT complex by prefoldin is well established. Experiments with archaeal prefoldin suggest that ternary complexes with substrate and CCT exist^44,45^. These complexes are unstable and prefoldin is released after substrate transfer to CTT before ATP binding and substrate folding by CCT complex^46,47^. It seems likely that Halo-HDAC1 might bind prefoldin and then form a ternary complex with CCT, but that subsequently either the process of prefoldin release or of ATP driven folding and release by CCT is interrupted (Fig. 7). This would prevent transfer of the active HDAC into functional HDAC complexes. Previous evidence is consistent with our hypothesis that prefoldin transfer of HDAC1 to CCT for processing occurs in the nucleus. Although nascent actin and tubulin amino acid polymers interact with prefoldin while still associated with the ribosome^37^, there is also evidence for a nuclear role for prefoldin (reviewed by Millan-Zambrano *et al*.^48^). Consistent with our observation of nuclear VBP1 in the presence of constitutively expressed HDAC1, VBP1 (prefoldin subunit 3) also relocalizes to the nucleus in the presence of another VBP1 interactor, VHL^49^. In addition, the prefoldin subunit, PFDN5/MM-1, colocalises with HDAC1 and Sin3A in the nucleus^50^. A role for nuclear CCT also has precedence; the TCP1 subunit is localized at heterochromatin in somatic cells in rats/mice^51^. In summary, although the evidence does not exclude initial binding of prefoldin to HDAC1 during translation, it is consistent with a nuclear role for HDAC1 processing by prefoldin and CCT.

The association of the CCT complex with HDAC1 may reflect the need to complete folding of a β sheet within HDAC1 prior to its use in active complexes. CCT mediated folding of β strands in other client proteins supports this. For example, CCT substrate VHL binds CCT via two β strands within the VHL β domain and this interaction appears to stabilize this domain during folding^40^. In addition, Yam *et al*. have suggested a high β sheet propensity globally among CCT client proteins^41^, as well as an enrichment in proteins that are components of multisubunit protein complexes among CCT substrates. They suggested that CCT could function to hold monomeric subunits in an assembly competent inactive state prior to assembly into complexes. Applied to HDAC1, such a model suggests a possible mechanism of preventing spurious deacetylation by free, newly synthesized HDAC1 molecules. Curiously, there is a β strand at the N terminus HDAC1 (shown in red in Fig. 7) that is folded into the HDAC1 β sheet (green), which could be a candidate CCT recognition site. The location of this strand near the N-terminus could explain why an N-terminal affinity tag might disrupt CCT processing of HDAC1.

In summary, we report that different affinity tagged versions of the histone deacetylase HDAC1 preferentially associate with either the prefoldin and chaperonin CCT (also known as TRiC) complexes and have low activity, or associate with the components of histone deacetylase complexes (Sin3, NuRD, CoREST and MiDAC) and possess high activity, depending on the location of the affinity tag. We propose that the N-terminal affinity tag on the recombinant Halo-HDAC1 protein allows it to associate with the CCT complex but prevents it being released from the CCT complex for assembly into nuclear histone deacetylase complexes. In contrast, we propose that C-terminally tagged HDAC1-Halo can be processed by CCT to generate an active enzyme that can be assembled into histone deacetylase complexes. As a result of these studies we propose a model where the prefoldin and CCT complexes play important roles in the assembly of active chromatin associated HDAC1 and HDAC2 complexes. Significantly, both CCT and HDAC complexes are potential druggable targets for cancer therapy. The HDAC inhibitor SAHA is currently used for treating triple negative breast cancer TNBC patients^52^, and the drug CT20p, which targets the CCT complex, is currently being investigated for its therapeutic potential for lung and breast cancer treatment^53,54^. Based on our results, it is conceivable that investigating therapeutic approaches that target both HDAC and CCT complexes together could be worthwhile.

## Materials and Methods

### Materials

Magne® HaloTag® beads (G7281) and TMRDirect™ fluorescent ligand (G2991) were from Promega. AcTEV protease (#12575015) was from Thermo Fisher Scientific. Clone FHC02563 containing the HDAC1 open reading frame was from the Kazusa DNA research institute (Kisarazu, Chiba, Japan). HeLa cells (ATCC^®^ CCL-2^™^) and HEK293T cells (ATCC^®^ CRL-11268^™^) were from American Type Culture Collection. Rabbit anti-SAP30 (ab125187) and rabbit anti-SUDS3 (ab184555) polyclonal antibodies were from Abcam. Rat anti-TCP1 (MA3-026) monoclonal antibody was from Life Technologies. Rabbit anti-HDAC1 (10197-1-AP) polyclonal antibody was from Proteintech. Mouse monoclonal anti-FLAG(M2) antibody (F3165) was from Sigma. IRDye® 800CW labeled goat anti-Rabbit (926-3211), IRDye® 680LT labeled goat anti-Mouse (926-68020) and IRDye® 680RD labeled goat anti-Rat (925-68076) secondary antibodies were from LI-COR Biosciences. Boc-Lys(Ac)-AMC was from ApexBio.

### Cloning sequences to express affinity tagged HDACs

HDAC1 was amplified from clone FHC02563 (Kazusa) using the primers listed in Supplementary Data and inserted into pFN21A or pFC14A. A codon optimized synthetic sequence coding for HDAC2 was subcloned into pFN21A (directly) or into pFC14A using the primers listed in Supplementary Data.

### Halo-HDAC1 stable cell line construction

The Flp-In™ System (Invitrogen) was used to generate a cell line expressing Halo-HDAC1 under the control of the CMVd2 promoter in Flp-In™-293 host cells essentially as described previously^55^.

### Preparation of whole cell lysates

Approximately 2 × 10^7^ HeLa cells or HEK293T cells were transiently transfected with 7.5 μg of plasmid DNA encoding Halo tagged versions of HDAC1 or HDAC2 as indicated in the figure legends. Forty-eight hours after transfection, cells were washed with PBS, harvested, and the resulting cell pellets were frozen at −80 degrees for at least 30 minutes. Cells were resuspended in 300 μl ice cold buffer containing 50 mM Tris·HCl (pH 7.5), 150 mM NaCl, 1% Triton^®^X-100, 0.1% sodium deoxycholate, 0.1 mM benzamidine HCl, 55 μM phenanthroline, 10 μM bestatin, 20 μM leupeptin, 5 μM pepstatin A, and 1 mM PMSF. The resulting lysates were passed through a 26-gauge needle 5 times, centrifuged at 21,000 × g for 30 minutes at 4 °C, and the resulting supernatant used for further analysis.

### Purification of HDAC complexes from human cells

Lysates were diluted by adding 700 μl TBS, centrifuged at 21,000 × g for 30 min at 4 °C and the supernatant was added to Magne® HaloTag® beads (Promega) prepared from 100 μl bead slurry according to the manufacturer’s instructions. The beads were mixed with the lysate using a tube rotator for at least two hours at 4 °C. Beads were then washed four times in buffer containing 25 mM Tris·HCl pH 7.4, 137 mM NaCl, 2.7 mM KCl and 0.05% Nonidet^®^ P40. Bound proteins were eluted using 100 μl buffer containing 50 mM Tris·HCl pH 8.0, 0.5 mM EDTA and 0.005 mM DTT, 2 Units AcTEV™ Protease (Thermo Fisher Scientific/Invitrogen) for 2 hours at 25 °C.

### Digestion of proteins for mass spectrometry

Purified proteins were precipitated by incubation with 20% trichloroacetic acid overnight at 4 °C. The precipitated proteins were concentrated by centrifugation, washed twice in ice-cold acetone, and residual acetone removed using a vacuum concentrator. Proteins were then resuspended in buffer containing 100 mM Tris·HCl pH 8.5 and 8 M urea. Disulphide bonds were reduced by adding 0.5 mM tris(2-carboxylethyl)-phosphine hydrochloride (TCEP) and incubating samples at room temperature for 30 minutes. Samples were treated with 10 mM chloroacetamide (CAM) for a further 30 minutes to prevent disulphide bond reformation. Denatured proteins were digested with endoproteinase Lys-C for at least 6 hours. The urea concentration was then reduced to 2 M using 100 mM Tris·HCl pH 8.5, CaCl_2_ was added to a final concentration of 2 mM and proteins further digested with trypsin overnight. Reactions were stopped by adding formic acid (5% final concentration).

### MudPIT mass spectrometry

Samples were pressure loaded onto three phase microcapillary columns (reversed phase/strong cation exchange/reversed phase). Bound peptides were eluted with a series of ten 2 hour MudPIT steps as previously described^56^ using a quaternary HPLC pump (Agilent) and analysed using a linear ion trap (LTQ) mass spectrometer in positive ion mode.

### Mass spectrometry data analysis

The RAW files were converted to.ms2 files using RAWDistiller v. 1.0. The ProLuCID algorithm version 1.3.5^57^ was used to match MS/MS spectra to a database containing human protein sequences, common contaminants and shuffled versions of all sequences (for estimating false discovery rates (FDRs)). Spectral and protein FDRs are listed in Supplementary Table S6. Searches were performed for peptides with static carboxamidomethylation modifications on cystine residues (+57 Daltons) and for peptides with dynamic oxidation modifications on methionine residues (+16 Daltons). An in-house software algorithm, swallow, was used in combination with DTASelect^58^ to filter out inaccurate matches. We have reported some of the mass spectrometry data used in this study previously: a summary of all mass spectrometry runs and details of where they were first reported is in Supplementary Table S6.

### Imaging

HEK293T cells were plated to 40% confluence in MatTek glass bottom culture dishes and cultured for 24 hours at 37 °C in 5% CO_2_. Cells were then transfected with plasmids expressing either Halo-HDAC1 or HDAC1-Halo. Halo-tagged proteins were labeled by adding HaloTag® TMRDirect™ ligand (20 nM final concentration). Cells were further cultured overnight and stained for 1 hour with Hoechst dye to label nuclei. Cells were washed twice with Opti-MEM® reduced serum medium and imaged with an LSM-700 Falcon confocal microscope. SNAP-tagged VBP1 was similarly imaged using SNAP-Cell® 505-Star ligand (NEB) according to the manufacturer’s instructions.

### HDAC activity assays

HDAC activity assays were performed essentially as described^59^ with minor modifications. Reactions containing 5 µl purified HDAC sample, 0.1 mM Boc-Lys(Ac)-AMC, 50 mM Tris·HCl pH 7.4, 137 mM NaCl, 2.7 mM KCl were incubated with or without 10 µM suberanilohydroxamic acid (SAHA) for 1 hour at 37 °C. Reactions were stopped by adding 10 µM SAHA and deacetylated substrate digested with trypsin (5 mg/ml) for 1 hour at 37 °C. Fluorescence was read using a SPECTRAmax GEMINI XS plate reader (excitation wavelength 355 nm, emission wavelength 460 nm).

## Electronic supplementary material

Supplementary Information

Supplementary Table 1

Supplementary Table 2

Supplementary Table 3

Supplementary Table 4

Supplementary Table 5

Supplementary Table 6

## Data Availability

Original data underlying this manuscript can be accessed from the Stowers Original Data Repository at http://www.stowers.org/research/publications/LIBPB-1235. The mass spectrometry datasets generated for this study are available from the Massive data repository (https://massive.ucsd.edu) using the identifiers listed in Supplementary Table S6. Additional datasets initially generated for a previous study^56^ and used for analysis in this study are also available from the PeptideAtlas data repository (http://www.peptideatlas.org/) using the identifiers listed in Supplementary Table S6.
