# Supplementary material for: Differential HDAC1/2 network analysis reveals a role for prefoldin/CCT in HDAC1/2 complex assembly
Source: Sci Rep. 2018 Sep 12;8:13712. doi: 10.1038/s41598-018-32009-w (PMC6135828; doi:10.1038/s41598-018-32009-w)
Supplement: Supplementary file 1 — Supplementary Information [file 41598_2018_32009_MOESM1_ESM.pdf]

Supplementary Data for

Differential HDAC1/2 network analysis reveals a role for prefoldin/CCT in HDAC1/2 complex assembly

Charles A. S. Banks[1], Sayem Miah[1], Mark K. Adams[1], Cassandra G. Eubanks[1], Janet L. Thornton[1], Laurence Florens[1], Michael P. Washburn[1,2]

[1]Stowers Institute for Medical Research, Kansas City, MO 64110 and [2]Department of Pathology & Laboratory Medicine, University of Kansas Medical Center, Kansas City, KS 66160

Index of Supplementary Data

Supplementary Data – DNA Sequences

1. Primers used in this study
2. Synthetic DNA sequence coding for HDAC2

Supplementary Figure 1: Western blot analysis of Halo-HDAC1/HDAC1-Halo expressed in HeLa cells.

Supplementary Figure 2: Full length gel images corresponding to figure 3D.

Supplementary Figure 3: Differential Interaction Network Analysis using Diffany.

Supplementary Figure 4: HDAC1 copurifies with SNAP-FLAG-VBP1 captured from HEK293T cell lysates.

Supplementary Figure 5: Full length gel images corresponding to figure 5B, Supplementary Figure 4 and Figure 6C.

Supplementary Tables:

Supplementary Table S1: Tagged HDAC1 and HDAC2 proteins used in a selection of published studies

Supplementary Table S2: HDAC1 associated proteins in HeLa cells

Supplementary Table S3: HDAC1 complexes in HeLa cells

Supplementary Tables S4: HDAC2 complexes in HeLa cells

Supplementary Tables S5: HDAC1 associated proteins in HEK293T cells

Supplementary Tables S6: Summary of mass spectrometry runs used in this study

Supplementary Data – DNA Sequences

1. Primers used to construct vectors expressing Halo tagged HDAC subunits

For cloning Halo-HDAC1 in pFN21A:

HDAC1 SgfI F 5'- CAGGCGATCGCCATGGCGCAGACGCAGG - 3'

HDAC1 PmeI R (with stop) 5'- CAGGTTTAAACTCAGGCCAACTTGACCTCCTC - 3'

For cloning HDAC1-Halo in pFC14A:

HDAC1 SgfI F (2) 5'- AAAAGCGATCGCCATGGCGCAGACG - 3'

HDAC1 PmeI R (no stop) 5'- AAAAGTTTAAACGGCCAACTTGACCTCC - 3'

For cloning HDAC2-Halo in pFC14A

HDAC2 SgfI F 5'- AAAAGCGATCGCCATGGCATATAGC - 3'

HDAC2 PmeI R (no stop) 5'- AAAAGTTTAAACCGGATTAGAGAGCTGCTCAG - 3'

2. Synthetic sequence used to express HDAC2 (subcloned into pFN21A)

5’ - GCGATCGCCATGGCATATAGCCAAGGTGGGGGCAAAAAAAAAGTATGTTATTATTATGA
TGGAGATATCGGTAACTATTACTATGGTCAGGGCCACCCAATGAAGCCACATAGAATCAGG
ATGACTCACAACCTGCTGTTGAATTATGGGTTGTATCGAAAGATGGAGATCTATAGACCACA
TAAGGCCACTGCTGAAGAGATGACCAAGTATCATTCTGATGAGTATATTAAATTTCTGAGGT
CCATCAGGCCAGACAACATGAGTGAGTACAGTAAACAAATGCAGCGCTTTAATGTGGGTGA
GGATTGCCCAGTGTTCGACGGTCTGTTTGAGTTCTGTCAGCTCAGCACTGGCGGCTCTGTTGC
AGGGGCGGTAAAACTTAACCGCCAGCAAACGGATATGGCTGTTAATTGGGCAGGTGGCTTG
CATCATGCGAAAAAGTCCGAGGCTAGTGGGTTTTGTTACGTCAACGATATTGTCTTGGCCAT
CCTTGAGTTGCTCAAATACCATCAGCGCGTTCTCTACATAGATATCGATATCCATCACGGTG
ACGGCGTGGAAGAGGCCTTTTACACAACAGACAGAGTGATGACGGTTTCCTTCCACAAGTAT
GGCGAGTATTTCCCTGGCACTGGCGATCTCCGCGACATCGGTGCGGGTAAAGGAAAGTACTA
CGCGGTTAACTTCCCCATGCGAGACGGGATAGACGACGAGTCCTATGGCCAAATATTTAAGC
CCATAATATCTAAAGTGATGGAAATGTATCAACCGAGCGCCGTGGTTTTGCAGTGCGGGGCA
GATAGTTTGTCTGGTGATCGCCTTGGGTGCTTTAATCTTACCGTCAAAGGTCACGCCAAGTGC
GTAGAAGTAGTCAAGACATTCAACTTGCCTCTCCTCATGTTGGGGGGCGGCGGGTACACGAT
CAGAAATGTGGCTAGATGTTGGACGTATGAAACCGCAGTTGCGCTGGATTGCGAGATTCCAA
ATGAGTTGCCTTACAACGATTATTTTGAATATTTTGGCCCGGATTTCAAACTTCATATTTCCC
CTAGTAATATGACTAACCAAAATACCCCGGAATATATGGAAAAAATAAAACAGCGACTCTT
CGAGAATCTTCGGATGTTGCCCCATGCTCCCGGCGTGCAGATGCAGGCGATACCAGAGGATG
CGGTCCACGAGGACAGCGGAGATGAAGATGGTGAGGACCCGGATAAGCGAATATCCATTCG
CGCCAGTGATAAACGGATAGCCTGTGACGAAGAATTTTCTGACAGTGAAGATGAGGGAGAG
GGAGGCCGCAGGAATGTGGCTGACCATAAAAAGGGCGCGAAGAAAGCAAGAATTGAAGAG
GACAAAAAGGAAACAGAGGACAAGAAGACTGATGTTAAGGAGGAGGACAAGTCAAAAGAC
AACAGTGGTGAGAAAACAGATACTAAAGGAACTAAATCTGAGCAGCTCTCTAATCCGTGAG
TTTAAAC - 3’

Supplementary Figure 1. Western blot analysis of Halo-HDAC1/HDAC1-Halo expressed in HeLa cells. Lysates from HeLa cells transiently transfected with plasmids expressing either Halo tag alone, Halo-HDAC1 or HDAC1-Halo were prepared as described in Materials and Methods. HDAC complexes were purified from lysates using Halo affinity purification as described in Materials and Methods. Samples of the HeLa cell lysates (left panel) or Halo purified protein complexes (right panel) were fractionated by SDS-PAGE and analysed by Western blotting using rabbit anti-HDAC1 primary antibodies and IRDye® 800CW labeled goat anti-Rabbit secondary antibodies. Proteins were visualised using an Odyssey CLx infra-red imaging system (LI-COR Biosciences).

Supplementary Figure 2. Full length gel images corresponding to figure 3D. Images are of Western blots probed with the indicated primary antibodies and fluorescently labeled secondary antibodies. Blots were scanned using a Li-Cor Odyssey infra-red scanner and visualised using the corresponding Li-Cor software Image Studio (version 2.1). Images were exported as .tiff files at 600 dpi.

Supplementary Figure 3. Constructing a differential protein interaction network using Diffany. To illustrate the rewiring of the HDAC1/2 interaction network after affinity tag relocation, we first created two separate interaction networks and then subtracted one network from the other using Diffany software. The initial networks mapped interactions of prey proteins with either N terminally or C terminally tagged versions of HDAC1/2 (network N or network C). Each interaction in these networks has an associated dBNSAF value. We next subtracted network C from network N to generate the differential interaction network. Edge widths (unbundled regions in Fiigure 4) represent differences in dBNSAF values with positive values in red and negative values in green.

Supplementary Figure 4. HDAC1 copurifies with SNAP-FLAG-VBP1 captured from HEK293T cell lysates. HEK293T cells were transiently transfected with plasmids expressing either Halo-HDAC1 or HDAC1-Halo with or without SNAP-FLAG-VBP1 as indicated. Lysates were prepared as described in Materials and Methods. SNAP-FLAG-VPB1 associated proteins were purified using SNAP affinity purification. Samples were fractionated by SDS-PAGE and analysed by Western blotting using rabbit anti-Halo primary antibodies and IRDye® 800CW labeled goat anti-Rabbit secondary antibodies (green bands),or using mouse anti-FLAG primary antibodies and IRDye® 680RD labeled goat anti-Mouse secondary antibodies (red bands). Proteins were visualised using an Odyssey CLx infra-red imaging system (LI-COR Biosciences). Full length images of Western blots are presented in Supplementary Figure 5.

Supplementary Figure 5. Full length gel images corresponding to figures 5B, Supplementary Figure 4, and Figure 6C. Western blots were probed with the indicated primary antibodies and fluorescently labeled secondary antibodies. Western blots and the Coomassie stained gel were scanned using a Li-Cor Odyssey infra-red scanner and visualised using the corresponding Li-Cor software Image Studio (version 2.1). Images were exported as .tiff files at 600 dpi.

Supplementary Figure 1: Western blot analysis of Halo-HDAC1/HDAC1-Halo expressed in HeLa cells

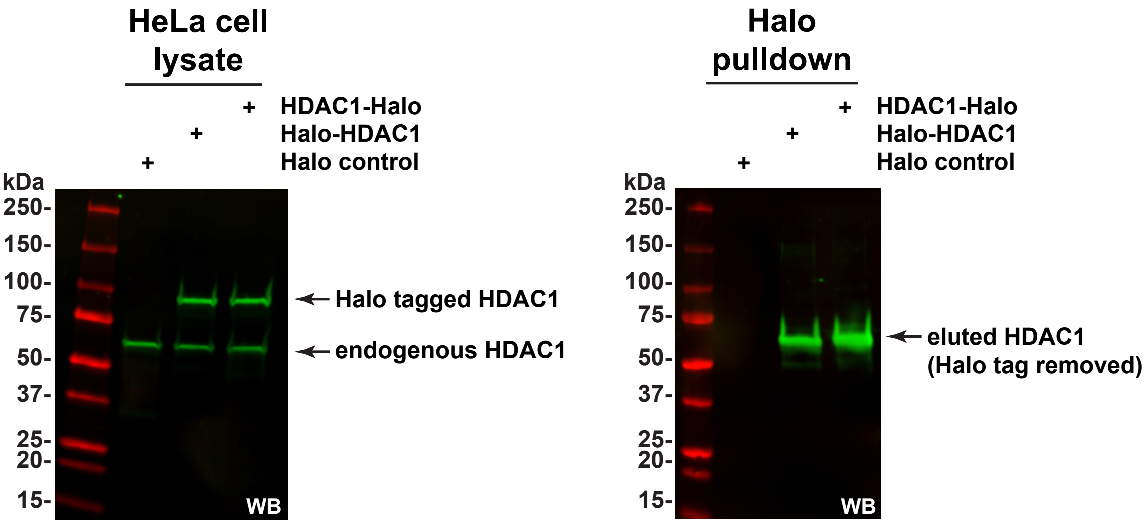

Lysates from HeLa cells transiently transfected with plasmids expressing either Halo tag alone, Halo-HDAC1 or HDAC1-Halo were prepared as described in Materials and Methods. HDAC complexes were purified from lysates using Halo affinity purification as described in Materials and Methods. Samples of the HeLa cell lysates (left panel) or Halo purified protein complexes (right panel) were fractionated by SDS-PAGE and analysed by Western blotting using rabbit anti-HDAC1 primary antibodies and IRDye® 800CW labeled goat anti-Rabbit secondary antibodies. Proteins were visualised using an Odyssey CLx infra-red imaging system (LI-COR Biosciences).

Supplementary Figure 2: Full length gel images corresponding to figures 3D

Figure 3D

Key:
- M Size markers
- 1 Halo control
- 2 Halo-HDAC1
- 3 HDAC1-Halo

293 cell lysate

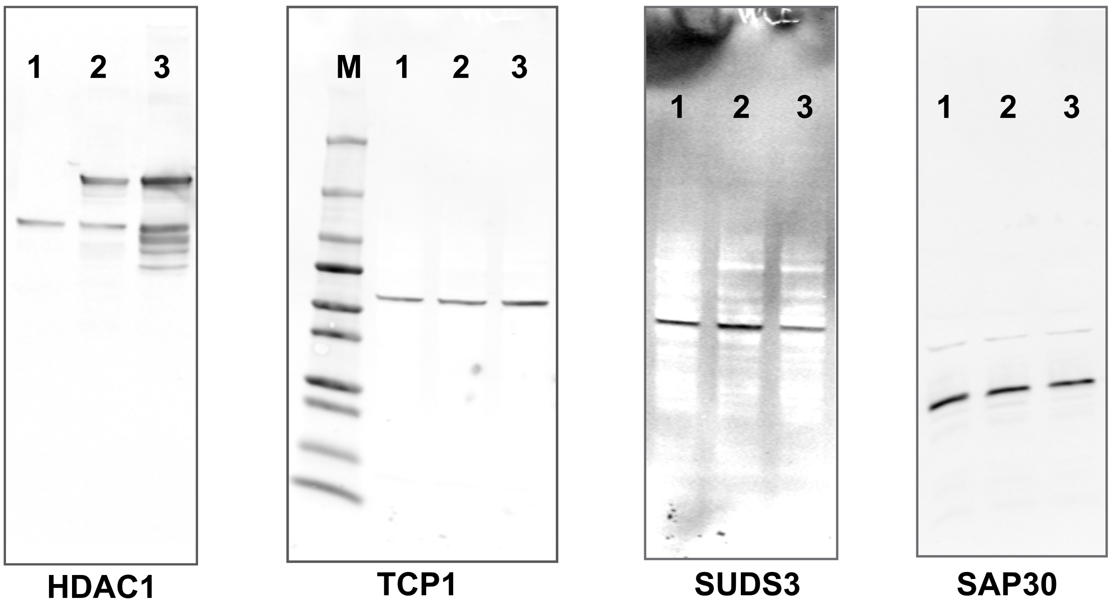

Halo Pulldown

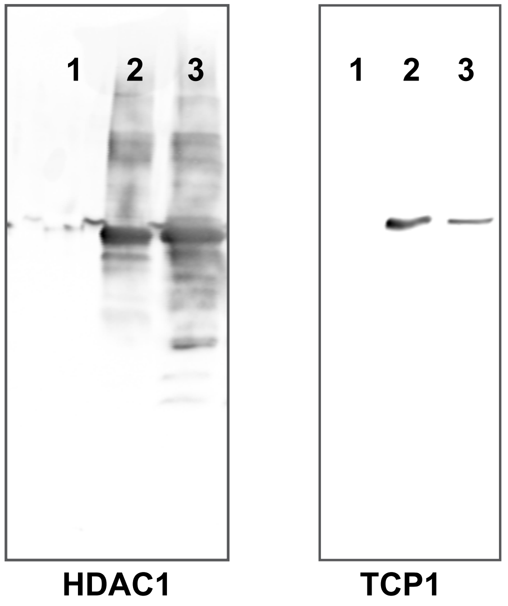

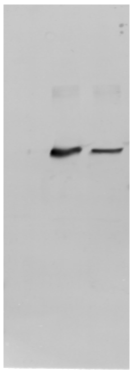

TCP1 Western blot with background signal increased abouve default settings using Image Studio (version 2.1)

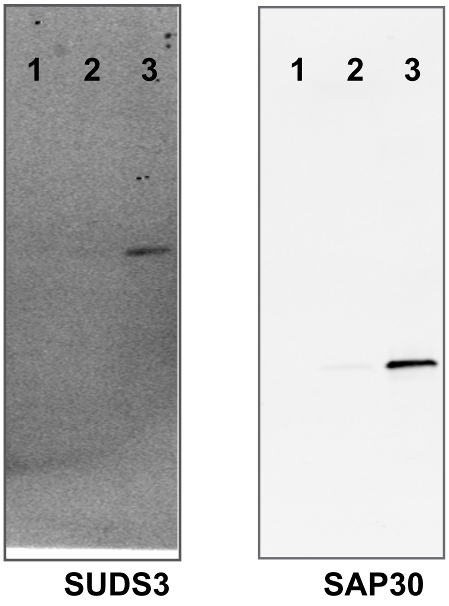

Images are of Western blots probed with the indicated primary antibodies and fluorescently labeled secondary antibodies. Blots were scanned using a Li-Cor Odyssey infra-red scanner and visualised using the corresponding Li-Cor software Image Studio (version 2.1). Images were exported as .tiff files at 600 dpi.

Supplementary Figure 3

Differential Interaction Network Analysis using Diffany[1]

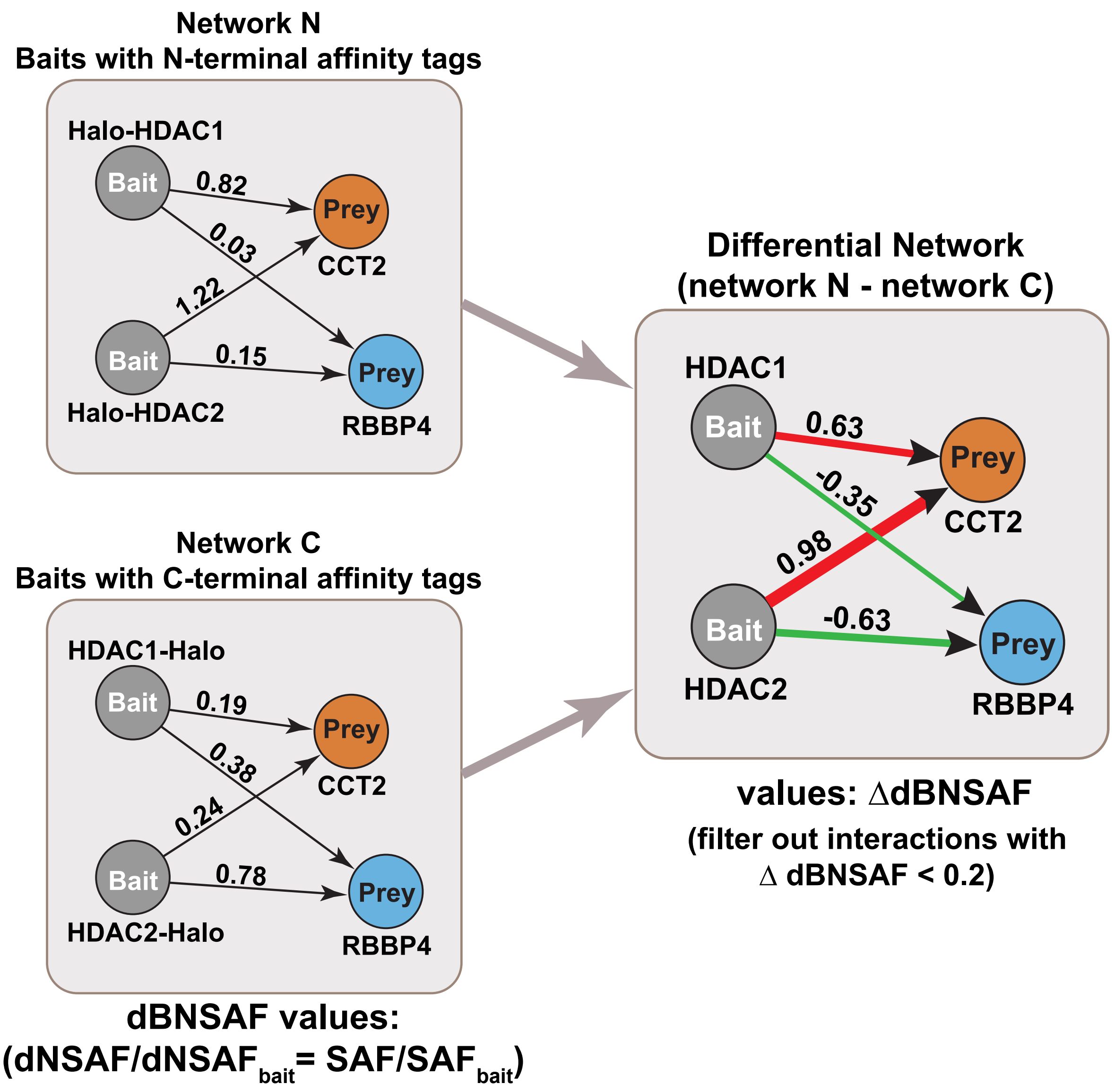

Contructing a differential protein interaction network using Diffany[1]–To illustrate the rewiring of the HDAC1/2 interaction network after affinity tag relocation, we first created two separate interaction networks and then subtracted one network from the other using Diffany software[1]. The initial networks mapped interactions of prey proteins with either N terminally or C terminally tagged versions of HDAC1/2 (network N or network C). Each interaction in these networks has an associated dBNSAF value.

We next subtracted network C from network N to generate the differential interaction network. Edge widths (unbundled regions in Fiigure 4) represent differences in dBNSAF values with positive values in red and negative values in green.

1. Landeghem, S. Van, Parys, T. Van, Dubois, M., Inzé, D. & Van De Peer, Y. Diffany: an ontology-driven framework to infer, visualise and analyse differential molecular networks. BMC Bioinformatics 17, 18 (2016).

Supplementary Figure 4:

HDAC1 copurifies with SNAP-FLAG-VBP1 captured from HEK293T cell lysates

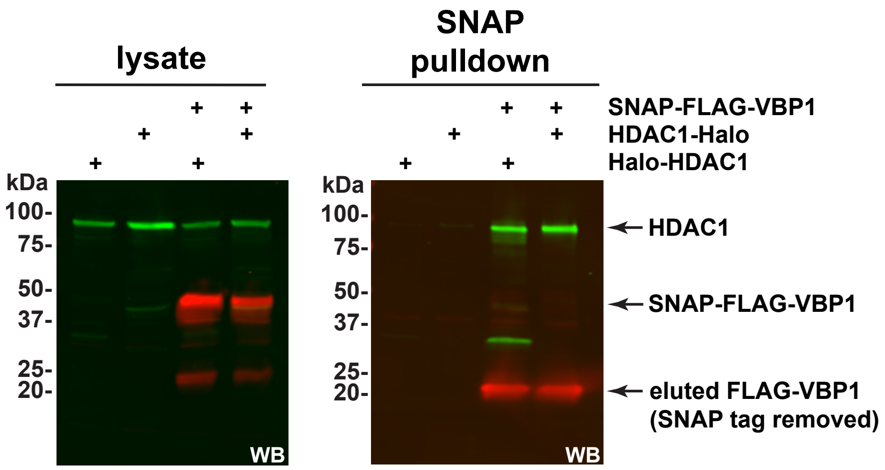

HEK293T cells were transiently transfected with plasmids expressing either Halo-HDAC1 or HDAC1-Halo with or without SNAP-FLAG-VBP1 as indicated. Lysates were prepared as described in Materials and Methods. SNAP-FLAG-VPB1 associated proteins were purified using SNAP affinity purification as described previously[1]. Samples were fractionated by SDS-PAGE and analysed by Western blotting using rabbit anti-Halo primary antibodies and IRDye® 800CW labeled goat anti-Rabbit secondary antibodies (green bands), or using mouse anti-FLAG primary antibodies and IRDye® 680RD labeled goat anti-Mouse secondary antibodies (red bands). Proteins were visualised using an Odyssey CLx infra-red imaging system (LI-COR Biosciences). Full length images of Western blots are presented in Supplementary Figure 5.

1. Banks, C. A. S. et al. A Structured Workflow for Mapping Human Sin3 Histone Deacetylase Complex Interactions Using Halo-MudPIT Affinity-Purification Mass Spectrometry. Mol. Cell. Proteomics 17, 1432–1447 (2018).

Supplementary Figure 5:
Full length gel images corresponding to Figure 5B, Supplementary Figure 4, and Figure 6C

Figure 5B

293 cell lysate | Halo Pulldown | Halo Pulldown

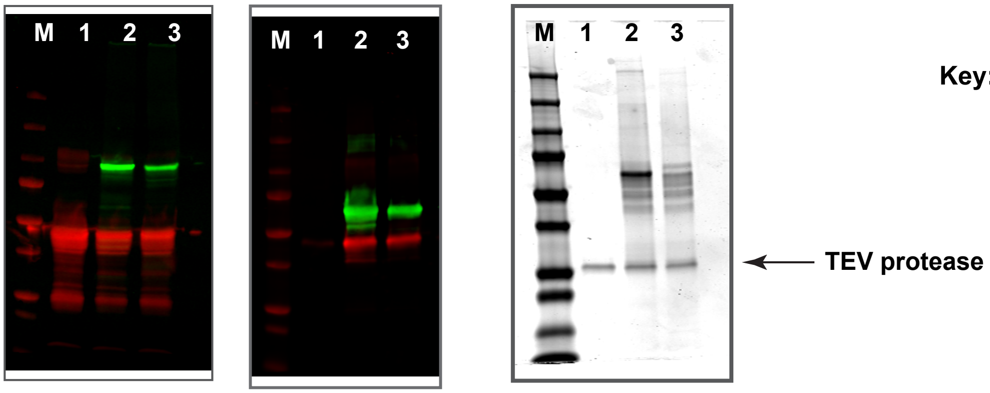

Western blots:
Coomassie stained markers (red)
anti FLAG (red)
anti-HDAC1 (green)

Coomassie stained gel

Key: M Size markers
1 SNAP-FLAG-VBP1
2 HDAC1-Halo + SNAP-FLAG-VBP1
3 Halo-HDAC1 + SNAP-FLAG-VBP1

Supplementary Figure 4

293 cell lysate | SNAP Pulldown

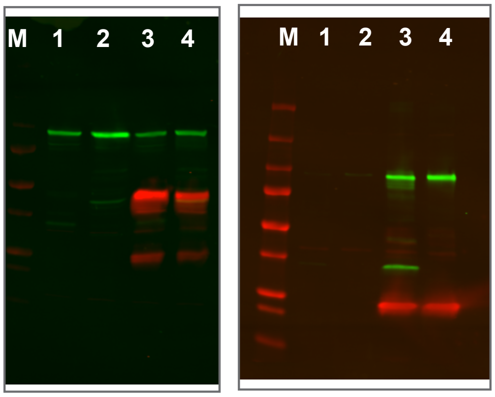

Key: M Size markers
1 Halo-HDAC1
2 HDAC1-Halo
3 SNAP-FLAG-VBP1 + Halo-HDAC1
4 SNAP-FLAG-VBP1 + HDAC1-Halo

Western blots:
Coomassie stained markers (red)
anti FLAG (red)
anti-Halo (green)

Figure 6C

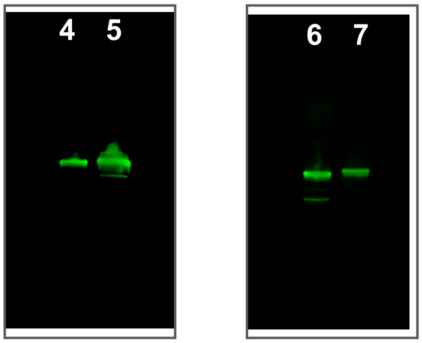

Key: 4 Halo-HDAC1
5 HDAC1-Halo
6 Halo-HDAC2
7 HDAC2-Halo

Western blots
lanes 4 and 5 anti-HDAC1 (green)
lanes 6 and 7 anti-HDAC2 (green)

Western blots were probed with the indicated primary antibodies and fluorescently labeled secondary antibodies. Western blots and the Coomassie stained gel were scanned using a Li-Cor Odyssey infra-red scanner and visualised using the corresponding Li-Cor software Image Studio (version 2.1). Images were exported as .tiff files at 600 dpi.
